# Sequence-based prediction of protein-protein interactions using weighted sparse representation model combined with global encoding

**DOI:** 10.1186/s12859-016-1035-4

**Published:** 2016-04-26

**Authors:** Yu-An Huang, Zhu-Hong You, Xing Chen, Keith Chan, Xin Luo

**Affiliations:** College of Computer Science and Software Engineering, Shenzhen University, Shenzhen, Guangdong 518060 China; School of Computer Science and Technology, China University of Mining and Technology, Xuzhou, Jiangsu 221116 China; Academy of Mathematics and Systems Science, Chinese Academy of Sciences, Beijing, 100190 China; Department of Computing, Hong Kong Polytechnic University, Kowloon, Hong Kong 999077 China

## Abstract

**Background:**

Proteins are the important molecules which participate in virtually every aspect of cellular function within an organism in pairs. Although high-throughput technologies have generated considerable protein-protein interactions (PPIs) data for various species, the processes of experimental methods are both time-consuming and expensive. In addition, they are usually associated with high rates of both false positive and false negative results. Accordingly, a number of computational approaches have been developed to effectively and accurately predict protein interactions. However, most of these methods typically perform worse when other biological data sources (e.g., protein structure information, protein domains, or gene neighborhoods information) are not available. Therefore, it is very urgent to develop effective computational methods for prediction of PPIs solely using protein sequence information.

**Results:**

In this study, we present a novel computational model combining weighted sparse representation based classifier (WSRC) and global encoding (GE) of amino acid sequence. Two kinds of protein descriptors, composition and transition, are extracted for representing each protein sequence. On the basis of such a feature representation, novel weighted sparse representation based classifier is introduced to predict protein interaction class. When the proposed method was evaluated with the PPIs data of *S. cerevisiae*, *Human* and *H. pylori*, it achieved high prediction accuracies of 96.82, 97.66 and 92.83 % respectively. Extensive experiments were performed for cross-species PPIs prediction and the prediction accuracies were also very promising.

**Conclusions:**

To further evaluate the performance of the proposed method, we then compared its performance with the method based on support vector machine (SVM). The results show that the proposed method achieved a significant improvement. Thus, the proposed method is a very efficient method to predict PPIs and may be a useful supplementary tool for future proteomics studies.

**Electronic supplementary material:**

The online version of this article (doi:10.1186/s12859-016-1035-4) contains supplementary material, which is available to authorized users.

## Background

Protein-protein interactions play a key role in various aspects of the functional organization of the living cell and take place in the signal transduction of any organism. Therefore, understanding PPIs is very important for the investigation of biological processes such as intercellular signaling pathways and modeling protein complex structures. Because of its significant status, the protein-protein interaction networks have been dawning increasing attention. Most of the protein-protein interaction data was collected by experimental methods like yeast two-hybrid (Y2H) screens [1, 2], tandem affinity purification (TAP) [3], mass spectrometric protein complex identification (MS-PCI) [4] and other high-throughput biological techniques for PPIs detection. A number of databases such as MINT [5], BIND [6] and DIP [7] have been established to store protein interaction information. However, these experimental methods are time-consuming and cost a lot. What’s worse, they can only identify a small number of interactions and fail to reach low rates of both false positive and false negative results. For these reasons, an increasing number of researchers are trying to develop a computational method for predicting PPIs.

Much effort has been devoted to propose computational approaches for detecting PPIs based on various data types, such as genomic information, protein domain and protein structure information. For example, Yu et al. [8] proposed a method based on secondary structures for inferring PPIs, and found that helix and disordered structures account for most of interacting regions. Similarly, Cai et al. [9] presented a SVM-based model which considers protein secondary structures, and yielded good prediction accuracy of 88.01 % when predicting PPIs of *Yeast* dataset. However, with the exponential growth of newly discovered protein sequences, there is a widening gap between the growing rate of protein sequences and that of protein structure data. For the sake of utilizing this wealth of protein sequence data, we develop effective sequence-based computational methods for predicting PPIs.

The existing computational methods for PPIs prediction from amino acid sequences mainly depend on the information of protein homology or interaction marks of the protein partners. Because of slow evolutionary divergence, homolog may still have the same or similar structures and functions. Based on this assumption, a number of methods based on the prior biological knowledge have been proposed. Zhao et al. [10] proposed a model based on position specific scoring matrix and auto covariance for predicting bioluminescent proteins and yield a high test accuracy of 90.71 %. Liu et al. [11] presented a protein feature extraction method considering the hydropathy profile of amino acids, and found it effectual for protein representation. However, these methods won’t work when detecting homolog with low sequence similarity. In addition, due to the similarity between the protein and its homolog decreases, it would be more difficult to use sequence homology recognition methods to solve the problem of predicting PPIs. Earlier studies [12] indicate that knowledge of the amino acid sequence alone might be sufficient to estimate the interacting propensity between two proteins. In this context, it is of great significance to develop computational methods by only using protein sequence information for predicting protein-protein interactions.

Current computational systems for predicting PPIs usually consist of two parts, feature extraction and machine learning model. As the first step of computational methods, feature extraction aims to mine useful information from original samples and represent them as normalized feature vectors of the same size. Effective feature extraction method usually helps the prediction system improve its performance. In this work, we adopt a method based on a global description of amino acid sequence and consider the physiochemical property of proteins in the process of feature extraction. This method would first classify 20 kinds of amino acids into 6 classes (e.g., C1 = {A, V, L, I, M, C}) and then gets 10 combinations each of which contains three different classes (e.g., {C1, C2, C3} vs {C4, C5, C6}). Based on these 10 combinations, a given protein sequence can be transformed into 10 binary characteristic sequences. Each characteristic sequences would be further divided into specific numbers of subsequences according to a partition method. Finally, two descriptors, composition and transition, would be extracted from these subsequences to depict the global composition of every protein sequence and form the final feature vectors.

Sample classification is the second step of computational models for predicting PPIs. Most of current computational methods are based on the traditional classifier such as support vector machine [13, 14] and neural network [15]. Although these classifiers have strong classification ability, they need much labor and time to adjust corresponding parameters for the best performance. Recently, sparse representation based classifier (SRC) is earning reputation for its powerful classification performance in the fields of signal processing, pattern recognition and computer vision. So it would be a good trial to explore it for building prediction systems for PPIs. Besides, SRC needs few parameters to adjust. In this study, we build a computational model by employing weighted sparse representation based classifier (WSRC), a variant of basic SRC, which integrates both sparsity and locality structure data into conventional SRC, and further improves the classification ability of SRC.

In this paper, we present a computational model for predicting PPIs by combining a novel global encoding representation of proteins and weighted sparse representation based classifier. We first adjusted the corresponding parameter (*L*) of global encoding method of protein sequence and transformed every protein sequence sample into a 150 dimensional vector. Secondly, we combined every two corresponding protein feature vectors into one representing a protein pair and then used these 300-dimensional vectors as the inputs for classifier. Finally, we adopted WSRC to classify the samples. We explored our proposed method to predict PPIs from three different dataset: *Yeast*, *Human* and *H. pylori*. To further estimate the performance of the proposed method, we compared it with the method based on the state-of-the-art classifier, support vector machine. In addition, in order to evaluate the generational ability of our proposed method, extensive experiments are performed to predict the PPIs from six other species datasets.

## Results

In this section, we firstly evaluate the performance of the proposed method for predicting three different datasets: *Yeast*, *Human* and *H. pylori* by using different evaluation measures including Receiver Operator Characteristic (ROC). We then compare the classification performances between WSRC and SVM by using the same feature extraction method. In addition, we also present the results of the experiments in which we used *Yeast* PPIs samples as training set to predict PPIs of other species datasets. Finally, we compare the performance of the proposed method with the previous existing methods.

### Evaluation measures

To evaluate the performance of the proposed method, we use the following criteria: the overall prediction accuracy (Accu.), sensitivity (Sens.), precision (Prec.) and Matthews’s correlation coefficient (MCC) were calculated. They are defined as follows:

1 $$ Accuracy=\frac{TP+TN}{TP+FP+TN+FN} $$

2 $$ Sensitivity=\frac{TP}{TP+FN} $$

3 $$ PE=\frac{TP}{TP+FP} $$

4 $$ MCC=\frac{TP\times TN-FP\times FN}{\sqrt{\left(TP+FN\right)\times \left(TN+FP\right)\times \left(TP+FP\right)\times \left(TN+FN\right)}} $$

where true positive (TP) denotes the number of true samples which are predicted correctly; false negative (FN) is the number of samples predicted to be non-interacting pairs incorrectly; false positive (FP) is the number of true non-interacting pairs predicted to be PPIs falsely, and true negative (TN) is the number of true non-interacting pairs predicted correctly. Furthermore, the ROC curve was also calculated to evaluate the performance of proposed method. Summarizing ROC curve in a numerical way, the area under an ROC curve (AUC) was computed.

### Parameter selection

For the sake of fairness, the corresponding parameters of weighted sparse representation based classifier would be set the same when explored in three different dataset—*Yeast*, *Human* and *H. pylori*. In this paper, we set σ = 1.5 and ε = 0.00005 when using the weighted sparse representation based classifier. As the parameter *L* is the unique parameter of the feature extraction method, the optimization of selection of *L* is of great importance for the model prediction performance. To search the best value of *L*, several experiments were performed by exploring *Yeast* PPIs dataset in the framework of 5-fold cross validation. The results are recorded in Table 1.

**Table 1:** Comparison among different L parameter values on *Yeast* dataset

L	Dimension	Acc. (%)	Prec. (%)	Sen. (%)	MCC (%)
4	120	96.09 ± 0.33	100.00 ± 0.00	92.18 ± 0.72	92.47 ± 0.62
5	150	96.82 ± 0.43	100.00 ± 0.00	93.63 ± 0.87	93.83 ± 0.81
6	180	96.66 ± 0.30	100.00 ± 0.00	93.32 ± 0.56	93.52 ± 0.56
8	240	96.39 ± 0.16	100.00 ± 0.00	92.78 ± 0.20	93.02 ± 0.28
12	360	96.28 ± 0.43	100.00 ± 0.00	92.57 ± 0.81	92.82 ± 0.80
16	480	96.16 ± 0.51	100.00 ± 0.00	92.32 ± 1.00	92.59 ± 0.95

It can be observed from Table 1 that the average accuracy gains an improvement reaching 96.82 % when *L* increases from 4 to 5. The reason is that, with a larger value of parameter *L*, GE descriptors can obtain more effective information. However, with the increase of the parameter *L*, the average prediction accuracy keeps a slight falling trend from 96.82 down to 96.16 %. The increase of *L* could also increase the complexity for computation, which may decrease the accuracy. Finally, we chose *L* = 5 in our experiments.

### Assessment of prediction ability

In order to evaluate the prediction ability of the proposed method, we explore *Yeast* and *H. pylori* dataset in this section. 5-fold cross validation is also used in our experiments in order to avoid the overfitting of the prediction model and test the performance stability. Specifically, one dataset was experimented for 5 times and we divided the whole dataset into five subsets in each time. Four of the subsets would take turns to be used for training and the rest one subset was used for testing. Here, we list the prediction results of the experiments in which we used the proposed model to predict PPIs of *Yeast* and *H. pylori* datasets (see Tables 2 and 3).

**Table 2:** 5-fold cross validation result obtained in predicting *Yeast* PPIs dataset

Test set	Accu.(%)	Prec.(%)	Sen.(%)	MCC(%)	AUC(%)
1	96.20	100.00	92.34	92.66	96.62
2	97.23	100.00	94.32	94.59	97.11
3	96.74	100.00	93.55	93.68	96.67
4	96.69	100.00	93.40	93.59	96.83
5	97.23	100.00	94.56	94.61	97.15
Average	96.82 ± 0.43	100.00 + 0.00	93.63 ± 0.87	93.83 ± 0.81	96.88 ± 0.24

**Table 3:** 5-fold cross validation result obtained in predicting *H. pylori* PPIs dataset

Test set	Accu.(%)	Prec.(%)	Sen.(%)	MCC(%)	AUC(%)
1	93.14	97.05	89.15	87.19	94.64
2	92.80	95.73	89.97	86.62	93.60
3	92.28	97.34	87.07	85.69	93.14
4	93.31	93.24	92.91	87.50	94.49
5	92.64	97.30	87.50	86.27	92.89
Average	92.83 ± 0.41	96.13 ± 1.75	89.32 ± 2.33	86.65 ± 0.72	93.75 ± 0.79

**Fig. 1:**
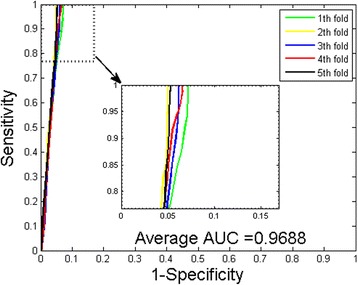
ROC from proposed method result for *Yeast* PPIs dataset

**Fig. 2:**
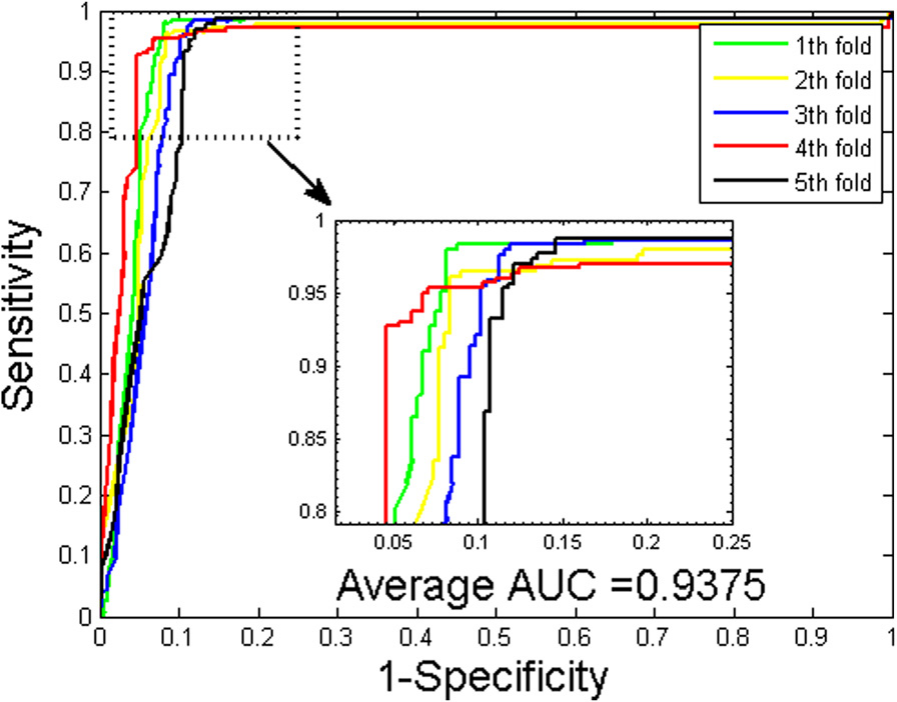
ROC from proposed method result for *H. pylori* PPIs dataset

It can be observed that when predicting the PPIs of *Yeast* dataset, the prediction accuracies are ≥96.20 %, the precisions are all 100 %, and the sensitivities are ≥93.63 %. Table 3 shows that when predicting the PPIs of *H. pylori* dataset, the prediction accuracies are ≥92.28 %, the precisions are ≥96.13 %, and the sensitivities are ≥89.32 %. Further, we can see that the standard deviations of these criteria are relative low. For the experiments on the *Yeast* dataset, the standard deviations of accuracy, precision and sensitivity are 0.43, 0.00 and 0.87 %. When exploring the *H. pylori* dataset, they come to be 0.41, 1.75 and 2.33 % respectively. To better quantify the prediction performance, Matthews correlation coefficient (MCC) and the AUC values of the ROC curves are also calculated. The averages of MCC and AUC values of experiments on *Yeast* dataset are 93.83 and 96.88 % respectively (see Fig. 1). For the experiments on *H. pylori* dataset, the proposed method yielded an average MCC and AUC value of 86.65 and 93.75 % (see Fig. 2).

The promising results show that the composition and transition descriptors in global encoding feature extraction sufficiently retain useful information from the original protein sequences. Considering the high accuracies and low standard deviations, the proposed method is feasible, effective and robust.

### Comparison with SVM-based method

There are various kinds of machine learning models having been proposed for predicting protein-protein interactions and one of the most prevalent classifiers is support vector machine (SVM). In order to evaluate the selected classification model of the proposed method, we further use support vector machine classifier to deal with an additional dataset, *Human* PPIs dataset, by using the same feature extraction method. A grid search method was used to optimize two corresponding parameters of SVM *c* and *g*. Here, we set *c* = 0.5 *g* = 0.5.

**Fig. 3:**
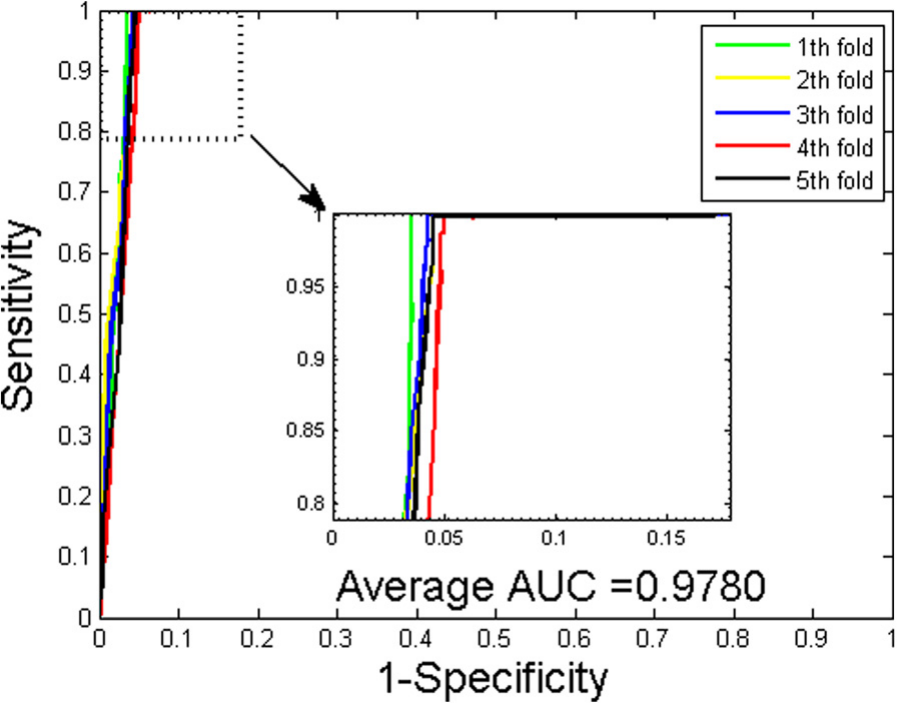
ROC from proposed method result for *Human* PPIs dataset

**Fig. 4:**
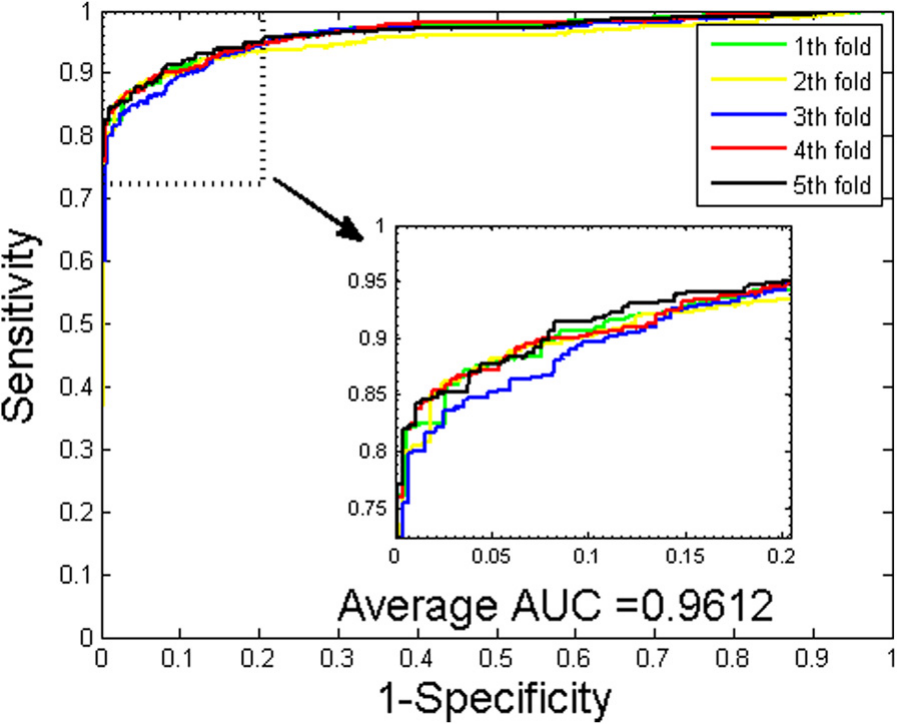
ROC from SVM-based method result for *Human* PPIs dataset

Table 4 shows the result comparison between WSRC and SVM classifier on *Human* dataset. It can be observed that WSRC yielded good results with averages of accuracy, precision, sensitivity and MCC as high as 97.66, 99.81, 95.28 and 95.41 % respectively. However, when using the SVM classifier, we obtained relatively poor results with the averages of accuracy, precision, sensitivity and MCC of 91.62, 97.05, 85.05 and 84.43 % respectively. The ROC curves of the experiments are also computed and shown in Figs. 3 and 4. It can be observed that the average AUC value performed by WSRC is 97.80 % higher than that performed by SVM classifier, which is 96.12 %. In addition, it should be noticed that the standard deviations of accuracy, precision, sensitivity and MCC yield by WSRC model are as low as 0.35, 0.12, 0.65 and 0.68 %, lower that those yield by SVM classifier which are 0.57, 0.59, 0.73 and 0.94 % respectively.

**Table 4:** 5-fold cross validation result obtained in predicting *Human* PPIs dataset

Classification model	Testing set	Accu.(%)	Prec.(%)	Sen.(%)	MCC(%)	AUC(%)
Proposed method	1	98.22	100.00	96.30	96.50	98.03
	2	97.73	99.73	95.47	95.54	98.17
	3	97.55	99.87	95.04	95.20	97.94
	4	97.30	99.73	94.61	94.72	97.30
	5	97.49	99.73	94.97	95.08	97.57
	Average	97.66 ± 0.35	99.81 ± 0.12	95.28 ± 0.65	95.41 ± 0.68	97.80 ± 0.36
SVM	1	91.79	96.70	85.84	84.75	96.43
	2	91.97	97.63	85.12	84.99	95.30
	3	90.63	96.21	83.86	82.78	95.90
	4	91.97	97.51	85.37	85.02	96.55
	5	91.73	97.20	85.05	84.60	96.44
	Average	91.62 ± 0.57	97.05 ± 0.59	85.05 ± 0.73	84.43 ± 0.94	96.12 ± 0.52

### Comparison with 2-MER feature extraction method

Different kinds of feature descriptors have been proposed for representing protein sequences. In this section, we further compare the performance of 2-MER feature descriptor with global encoding. 2-MER is a typical feature descriptor which records the frequencies of substrings of length 2. Specifically, we combine 2-MER descriptor with WSRC to predict the PPIs of *H. pylori* dataset in the frame work of 5-fold cross validation. For fair evaluation, the parameters were set to be the same as other experiments in this work (σ = 1.5 and ε = 0.00005).

**Fig. 5:**
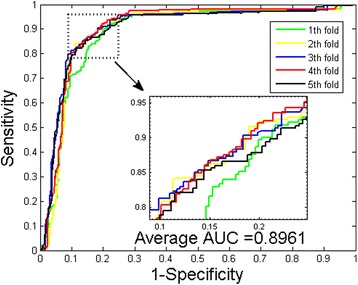
ROC yielded by combining 2-MER and WSRC

The comparison results are listed in Table 5. We can see that 2-MER feature extraction yielded relatively poor results with averages of accuracy, precision, sensitivity and MCC of 84.88, 83.23, 87.40 and 74.27 % respectively. For further evaluation, the ROC curves and AUC values are also computed. (see Fig. 5 and Table 5). The average AUC value yielded by adopting 2-MER feature extraction method was 89.61 %, lower than that yielded by the proposed model.

**Table 5:** Experimental results yielded by combing 2-MER and WSRC on *H. pylori* dataset

Classification model	Testing set	Accu.(%)	Prec.(%)	Sen.(%)	MCC(%)	AUC(%)
2-MER with WSRC	1	82.85	82.32	85.05	71.50	88.53
	2	86.79	86.88	85.96	77.06	89.32
	3	85.25	81.67	89.75	74.78	90.32
	4	86.11	86.69	88.05	75.83	90.13
	5	83.39	78.62	88.19	72.20	89.74
	Average	84.88 ± 1.71	83.23 ± 3.53	87.40 ± 1.88	74.27 ± 2.37	89.61 ± 071
Proposed model	Average	92.83 ± 0.41	96.13 ± 1.75	89.32 ± 2.33	86.65 ± 0.72	93.75 ± 0.79

### Performance on independent dataset

As the proposed model yielded good performance on the PPIs data of *Yeast*, *Human* and *H. pylori*, extensive computational analyses were performed in which we explored our method on six datasets of other species. In these experiments, we used all 11188 samples of yeast PPIs dataset for training and six different PPIs dataset of other species were used for testing. Here, parameters σ and ε were set to be 1.5 and 0.00005, the same as prior experiments. When predicting the PPIs on datasets of *D. mela*, *E. coli*, *C. elegans*, *H. sapien*, *H. pylori* and *M. musculus*, the accuracies are 89.35, 72.92, 88.99, 88.81, 85.77 and 83.39 % respectively. (see Table 6) Predicting five of these species, we obtained promising results with average accuracies of over 83 % while *E. col*i dataset got a relatively low accuracy which still reaches 72.92 %. When predicting the PPIs of datasets of *D. mela*, *C. elegans* and *H. sapien*, the accuracy even reach ≥88.8 %.

**Table 6:** Prediction results on five species based on our model

Species	Test pairs	Accuracy
*D. mela*	21975	89.35 %
*E. coli*	6954	72.92 %
*C. elegans*	4013	88.99 %
*H. sapien*	1412	88.81 %
*H. pylori*	1420	85.77 %
*M. musculus*	313	83.39 %

### Comparison with other methods

Various kinds of computational methods have been proposed for predicting PPIs. To further evaluate the performance of the proposed method for predicting protein interactions, we compare it with the existing methods on *Yeast* and *H. pylori* datasets. Table 7 shows the results performed by six other existing methods on *Yeast* dataset and it can be observed that none of these methods obtains better result than that performed by the proposed method, which yielded the highest average accuracy of 96.82 %. In addition, considering the relatively low standard deviations of accuracy, precision, and sensitivity which are 0.43, 0.00 and 0.87 %, the proposed method is more stable than the other existing methods. Table 8 shows the results performed by other five existing methods on *H. pylori* dataset. The accuracies yielded by other methods are between 75.80 and 86.60 %, all of whom are lower than 92.83 %, the accuracy performed by the proposed method.

**Table 7:** Performance comparison of different methods on the *Yeast* dataset

Model	Test set	Accu.(%)	Prec.(%)	Sen.(%)	MCC(%)
Guos’ work [35]	ACC	89.33 ± 2.67	88.87 ± 6.16	89.93 ± 3.68	N/A
	AC	87.36 ± 1.38	87.82 ± 4.33	87.30 ± 4.68	N/A
Zhous’ work [36]	SVM + LD	88.56 ± 0.33	89.50 ± 0.60	87.37 ± 0.22	77.15 ± 0.68
Yangs’ work [37]	Cod1	75.08 ± 1.13	74.75 ± 1.23	75.81 ± 1.20	N/A
	Cod2	80.04 ± 1.06	82.17 ± 1.35	76.77 ± 0.69	N/A
	Cod3	80.41 ± 0.47	81.86 ± 0.99	78.14 ± 0.90	N/A
	Cod4	86.15 ± 1.17	90.24 ± 1.34	81.03 ± 1.74	N/A
Proposed method	WSRC	96.82 ± 0.43	100.00 + 0.00	93.63 ± 0.87	93.83 ± 0.81

**Table 8:** Performance comparison of different methods on the *H. pylori* dataset

Model	Accu.(%)	Prec.(%)	Sen.(%)	MCC(%)
Phylogenetic booststrap [38]	75.80	80.20	69.80	N/A
HKNN [39]	84.00	84.00	86.00	N/A
Signature products [28]	83.40	85.70	79.90	N/A
Ensemble of HKNN [40]	86.60	85.00	86.70	N/A
Boosting [41]	79.52	81.69	80.37	70.64
Proposed method	92.83	96.13	89.32	86.65

## Discussion

The feature extraction of the proposed model is mainly based on the assumption that whether two proteins interact can be greatly influenced by their physicochemical characteristics such as residues’ hydrophobic property and charged property [16–21]. Adopting the concept of Local Binary Patterns (LBP), Global encoding uses a binary mapping strategy and global description to retain the information of physicochemical characteristics as well as the protein sequence information [22–26]. Two kinds of feature descriptor, composition and transition, are proposed based on this binary mapping. Composition descriptor aims to retain the distribution information and transition descriptor is used for recording the neighbour influence. To appropriately combine with global encoding which refers to the concept of LBP, we use a state-of-the-art classifier in the field of face recognition, WSRC, in the second step of model design.

It is worthwhile to highlight several aspects of the proposed approach here: (1) Based on the results of comparison experiments, we consider the selected classification method, WSRC, superior to the SVM classifier with higher accuracy and better stability. There are two possible reasons for good performance of our selected classification model. One reason lies in the fact that weighted sparse representation based classifier integrates both sparsity and locality structure data into conventional SRC, which help improve the prediction performance dealing with the global encoding descriptor. The similarity of global encoding to LBP descriptor may explain the superiority of WSRC, and Vapnik-Chervonenkis dimension of WSRC which is larger than SVM may lead to a fit with the global encoding descriptor. In addition, WSRC needs little manual intervention to adjust its corresponding parameters, which help us obtain good results without much effort. (2) Interestingly, the outstanding results of Table 5 show that yeast PPIs data is possibly sufficient for predicting PPIs of other species and that our proposed method is has a strong generational ability and powerful to deal with cross-species PPIs prediction. (3) It is known that ensemble classifier usually achieves more accurate and robust performance than the methods using single classifier. However, when predicting PPIs of *Yeast* and *H. pylori* dataset, our proposed model even yields a better result than some of existing method which are based on ensemble classifier such as boosting and ensemble of HKNN. From these comparisons, we consider the WSRC-based model combined with global encoding feature extraction method can significantly improve the prediction accuracy. (4) Global encoding retains the information of physicochemical characters and 2-MER descriptor doesn’t. Therefore, global encoding is expected to be superior to 2-MER for predicting PPIs and the results of comparison experiment conform to this anticipation. The results illustrate that physicochemical characters can help improve the performance for predicting PPIs.

## Conclusions

In order to obtain more knowledge on protein-protein interactions, developing effective computational methods for PPIs prediction become increasing important. In this work, we explore a novel prediction model for PPIs by combing weighted sparse representation based classifier and global encoding representation of proteins. In the process of feature extraction, two kinds of descriptors, composition and transition, are extracted from subsequences of global encoding. Weighted sparse representation based classifier would be finally used to deal with sample classification. The proposed method performs well when predicting on no matter one species data or cross-species data. Good results imply that our proposed method is feasible, superior and robust.

## Methods

### Gold standard datasets

We verify the proposed method on a high confidence Saccharomyces cerevisiae PPIs data set. This dataset is gathered from publicly available database of interacting proteins (DIP). The protein pairs which have ≥40 % sequence identity or whose lengths are less than 50 residues were removed. Consequently, we got the remaining 5594 protein pairs and used them to construct the positive data set. For the negative dataset, we chose 5594 additional protein pairs of different sub-cellular localizations. By doing this, the whole data set is made up of 11188 protein pairs of which half are from the positive samples and half are from the negative samples.

To demonstrate the generality of the proposed method, we also verify our approach on two other types of PPIs data sets. We collected the first dataset from the Human Protein References Database (HPRD). Those protein pairs which have ≥25 % sequence identity were removed. Finally, to comprise the golden standard positive dataset, we used the remaining 3899 protein-protein pairs of experimentally verified PPIs from 2502 different human proteins. For gold standard negative dataset, following the previous work [27], we assume the proteins in different subcellular compartments do not interact with each other and finally obtained 4262 protein pairs from 661 different human proteins as the negative dataset. As a result, the *Human* dataset is constructed by 8161 protein pairs. The second PPI dataset is constructed by 2916 helicobacter pylori protein pairs (1458 interacting pair and 1458 non-interacting pairs) as described by Martin et al. [28].

### Global encoding (GE) of amino acid sequence

The feature extraction method used in this work will be described in this section. Protein sequences would be first changed into ten binary sequences in a novel way and then we use two kinds of descriptors to extract features from these numerical sequences considering the distribution of all kinds of residues. To visually explain the process of this method, we give a simple example for illustration in Additional file 1: Figure S1. Global encoding (GE) of protein sequences could be obtained by the following steps.

#### Step 1. Transformation of protein sequence

Researches [29, 30] have pointed out that amino acids can be classified into 6 different classes according to the physicochemical characteristic such as residues’ hydrophobic property, charged property and so on (see Table 9). For the reduction of data complexity, we first encode the protein sequence substituting every amino acid by its class accordingly, and the substitution rules are presented in Table 10.

**Table 9:** Amino acid classification

Amino acid classification	
Aliphatic amino acid:	C1 = {A,V,L,I,M,C}
Aromatic amino acid:	C2 = {FW,Y}
Polar amino acid:	C3 = {S,TN,Q}
Positive amino acid:	C4 = {K,R}
Negative amino acid:	C5 = {D,E}
Special conformations:	C6 = {G,P}

**Table 10:** Example for the process of descriptors’ extraction

Subsequence:	1 0 1 0 0 1 1 1 1 0 0 1 1 0 1 0 1 0 1 0 1 0 1 1 0 0 1 1
Position of ‘0’:	0 0 0 0 0 0 0 0 0 0 0 0
Position of ‘1’:	1 1 1 1 1 1 1 1 1 1 1 1 1 1 1 1
‘1-0’ transition:	1 0 1 0 1 0 1 0 1 0 1 0 1 0 1 0 1 0 0
‘0-1’ transition:	0 1 0 1 0 1 0 1 0 1 0 1 0 1 0 1 0 1

In this way, every protein sequence is represented by six symbols: *C1*, *C2*…*C6*. Based on this classification, we can further divide these 6 classes into 2 subsets each of which contains 3 different classes. By doing this, ten modes can be obtained as follows: *{C1, C2, C3}* vs *{C4, C5, C6}*, *{C1, C2, C4}* vs *{C3, C5, C6}*, *{C1, C2, C5}* vs *{C3, C4, C6}*, *{C1, C2, C6}* vs *{C3, C4, C5}*, *{C1, C3, C4}* vs *{C2, C5, C6}*, *{C1, C3, C5}* vs *{C2, C4,C6}*, *{C1, C3, C6}* vs *{C2, C4, C5}*, *{C1, C4, C5}* vs *{C2, C3, C6}*, *{C1, C4, C6}* vs *{C2, C3, C5}* and *{C1, C5, C6}* vs *{C2, C3, C4}*. We then transform every protein sequence into ten binary sequences based on these ten modes correspondingly. Given a protein sequence *P* = *p*_*1*_*, p*_*2*_*,…,p*_*n*_, let’s symbolize the ten transformed sequences of *P* as *S*_*1*_*, S*_*2*_*,…, S*_*10*_. Here we enumerate the first two numerical sequences, *S*_*1*_*(p*_*i*_*)* and *S*_*2*_*(p*_*i*_*)*, as Eqs. (1) and (2) respectively:

5 $$ {S}_1\left({p}_i\right)=\left\{\begin{array}{l}\begin{array}{cc}\hfill 1\hfill & \hfill {p}_i\in \left\{{A}_1,{A}_2,{A}_3\right\}\hfill \end{array}\\ {}\begin{array}{cc}\hfill 0\hfill & \hfill {p}_i\in \left\{{A}_4,{A}_5,{A}_6\right\}\hfill \end{array}\end{array}\right.\kern0.5em \mathsf{i}=\mathsf{1}\dots \mathsf{n} $$

6 $$ {S}_2\left({p}_i\right)=\left\{\begin{array}{l}\begin{array}{cc}\hfill 1\hfill & \hfill {p}_i\in \left\{{A}_1,{A}_2,{A}_4\right\}\hfill \end{array}\\ {}\begin{array}{cc}\hfill 0\hfill & \hfill {p}_i\in \left\{{A}_3,{A}_5,{A}_6\right\}\hfill \end{array}\end{array}\right.\kern0.5em \mathsf{i}=\mathsf{1}\dots \mathsf{n} $$

Where *p*_*i*_ is the i-th amino acid of the given protein sequence. Here we call *S*_*i*_ as the i-th characteristic sequence.

#### Step 2. Partition of characteristic sequences

In this step, every characteristic sequences are further divided into subsequences of different lengths by a special strategy. For any characteristic sequence *S*_*n*_ = *s*_*1*_, *s*_*2*_,…,*s*_*n*_ of length *n*, given a positive integer *L*, *S*_*n*_ will be divided into *L* subsequences. We call the kth subsequence as *SubS*_*k*_ (*k* = *1*, *2*,…, *L*) and *SubS*_*k*_ is composed of the first ⌊*kn*/*L*⌋ numbers of *S*_*n*_. Here we present an example to explain the process of characteristic sequence partition in Table 11. In this sample, the length of the given sequence is 57 and parameter *L* is set to be 6. So the length of its subsequences is 9, 19, 28, 39, 47 and 57 respectively.

**Table 11:** Example for characteristic sequence partition

	Sequence:	Length
S_n_:	101001111001101010101011001101011010010110110101000100010	57
SubS_1_:	101001111	9
SubS_2_:	1010011110011010101	19
SubS_3_:	1010011110011010101010110011	28
SubS_4_:	10100111100110101010101100110101101001	38
SubS_5_:	10100111100110101010101100110101101001011011010	47
SubS_6_:	101001111001101010101011001101011010010110110101000100010	57

#### Step 3. Extraction of feature vectors

In the last step, feature vectors of composition and transition descriptors will be extracted from the subsequences produced in the prior step. The composition descriptor describes the frequencies of ‘0’ and ‘1’ in each subsequence. As a composition descriptor of one subsequence contains two frequency values, any characteristic sequence would be represented by a 2*L dimensional feature vector by the composition descriptor. Transition, as the second descriptor, account for the switch frequency between ‘0’ and ‘1’ in every subsequence. The times where ‘0’ follows 1’ and ‘1’ follows ‘0’ happen are counted independently. Here, we illustrate this method with the example in Fig. 1.

Table 10 shows the process of descriptors’ extraction from the subsequence 3 in the Table 11. The length of example sequence is 28; the numbers of ‘0’ and ‘1’ are 12 and 16 respectively; the transition times of ‘1-0’ and ‘0-1’ are both 9. Therefore, two values of composition descriptor are 12/28 = 42.86 % and 16/28 = 57.14 % respectively. The value of transition descriptor is 9 + 9 = 18. In this work, *L* is set to be 5 after adjusting for the best performance. As a protein sequence would be first transformed into 10 numerical sequences and each sequence would further be partitioned by 5 subsequences which can be represented by 3-dimension feature descriptors, the length of the whole feature vector of a protein sequence is 10*5*3 = 150.

### Weighted sparse representation based classification (WSRC)

In the recent years, major developments have taken place in compressed sensing (CS) theory and linear representation methods (LRBM). Based on these progresses, sparse representation is earning increasing attention in fields of signal processing, computer vision and pattern recognition. In the sparse representation based classification (SRC) [31], it is assumed sufficient to represent a given test sample by samples from the sample subject. Based on this theory, sparse representation based classifier try to use a sparse representation matrix to reveal this relation between the test sample and the whole training set. In SRC, the sparse representation matrix needs to be optimized. After obtaining this matrix and calculating the reconstruction residuals of each class, the test sample will be finally assigned to the class with the minimum reconstruction residual. To specifically explain the process of WSRC, we give a simple example for illustration in Additional file 2: Figure S2. Given a training set matrix *X* ∈ *R*^*m* × *n*^ representing n m-dimension training samples, SRC suppose that there are sufficient training samples belonging to *kth* class. The *kth* class samples can construct a sub matrix ${X}_k=\left[{l}_{k1},{l}_{k2}\dots {l}_{k{n}_k}\right]$ where *li* denotes the class of *ith* sample and *n*_*k*_is the number of sample belonging to *kth* class. So *X* can be further rewritten as *X =* [*X*_*1*_*X*_*2*_*… X*_*K*_] where *K* denotes the class number of the whole samples. Given a test sample, *y*ϵ*R*^*m*^, SRC represents it with the linear combination of training samples of k-th class:

7 $$ y={\alpha}_{k,1}{l}_{k,1}+{\alpha}_{k,2}{l}_{k,2}+\cdots +{\alpha}_{k,{n}_k}{l}_{k,{n}_k} $$

which can be further symbolized with the consideration of the whole training set representation as follow:

8 $$ y=X{\alpha}_0 $$

where ${\alpha}_0={\left[0,\cdots, 0,{\alpha}_{k,1},{\alpha}_{k,2}\cdots {\alpha}_{k,{n}_k},0,\cdots, 0\right]}^T$. For the reason that the nonzero entries in α0 are only associated with the *kth* class, when the class number of samples is large, *α*_*0*_would come to be sparse. The key of SRC algorithm is to search the *α*vector which can not only satisfy Eq. (8) but also minimize the l _0_-norm of itself:

9 $$ {\widehat{\alpha}}_0= \arg \min {\left\Vert \alpha \right\Vert}_0\kern0.5em \mathrm{subject}\ \mathrm{t}\mathrm{o}\kern0.5em y=X\alpha $$

Problem (9) is NP-hard problem which can be achieved but hardly to be solved precisely. Theory of compressive sensing [32, 33] shows that, when α is sparse enough, it is feasible to solve the related convex l_1_-minimization problem instead solving the solution of l_0_-minimization problem directly:

10 $$ {\widehat{\alpha}}_1= \arg \min {\left\Vert \alpha \right\Vert}_1\kern0.5em \mathrm{subject}\ \mathrm{t}\mathrm{o}\kern0.5em y=X\alpha $$

Dealing with occlusion, the Eq. (10) should be extended to the stable l_1_-minimization problem:

11 $$ {\widehat{\alpha}}_1= \arg \min {\left\Vert \alpha \right\Vert}_1\kern0.5em \mathrm{subject}\ \mathrm{t}\mathrm{o}\kern0.5em \left\Vert y-X\alpha \right\Vert \le \varepsilon $$

where*ε* > 0 denotes to the tolerance of reconstruction error. Given the solution from Eq. (11), the SRC algorithm assigns the label of test sample *y* to class *c* based on the following rule:

12 $$ \underset{c}{ \min }{r}_c(y)=\left\Vert y-X{\widehat{\alpha}}_1^c\right\Vert,\ \mathrm{c} = 1\dots \mathrm{K} $$

Lu et al. [34] have recently proposed a variant of traditional sparse representation based classifier called weighted sparse representation based classifier (WSRC). When dealing with classification problems, Nearest Neighbor (NN) classifier considers the influence of the nearest neighbor in the training set while SRC consider the linearity structure of data. Researches have shown that locality is more essential than sparsity in some case. For this reason, weighted sparse representation based classifier (WSRC) integrates the locality structure of data into basic sparse representation. Specifically, WSRC would first compute the Gaussian distance between the sample and the whole training samples and use these distances as the weights of each training samples. The Gaussian distance between two samples, s_1_ and s_2_, can be described as follow:

13 $$ {d}_G\left({s}_1,{s}_2\right)={e}^{-{\left\Vert {s}_1-{s}_2\right\Vert}^2/2{\sigma}^2} $$

where σ means the Gaussian kernel width. By this way, the locality structure of data can be retained. WSRC would then turn to solve the following problem:

14 $$ {\widehat{\alpha}}_1= \arg \min {\left\Vert W\alpha \right\Vert}_1\kern0.5em \mathrm{subject}\ \mathrm{t}\mathrm{o}\kern0.5em y=X\alpha $$

and specifically,

15 $$ diag(W)={\left[{d}_G\left(y,{x}_1^1\right),\dots, {d}_G\left(y,{x}_{n_k}^k\right)\right]}^T $$

where *W* is a block-diagonal matrix of locality adaptor and *n*_*k*_ is the sample number of training set in class *k*. Dealing with occlusion, we would finally solve the following stable l_1_-minimization problem:

16 $$ {\widehat{\alpha}}_1= \arg \min {\left\Vert W\alpha \right\Vert}_1\kern0.5em \mathrm{subject}\ \mathrm{t}\mathrm{o}\kern0.5em \left\Vert y-X\alpha \right\Vert \le \varepsilon $$

where *ε* > 0 is the tolerance value.

The WSRC algorithm can be summarized as following steps:

## Additional files

Additional file 1: Figure S1. Example for illustrating the process of global encoding (TIF 781 kb) Additional file 2: Figure S2. Example for illustrating the process of weighted sparse representation based classifier. (TIF 1088 kb)
