# Supplementary figures and images for: Sequence-based prediction of protein-protein interactions using weighted sparse representation model combined with global encoding
Source: BMC Bioinformatics. 2016 Apr 26;17:184. doi: 10.1186/s12859-016-1035-4 (PMC4845433; doi:10.1186/s12859-016-1035-4)

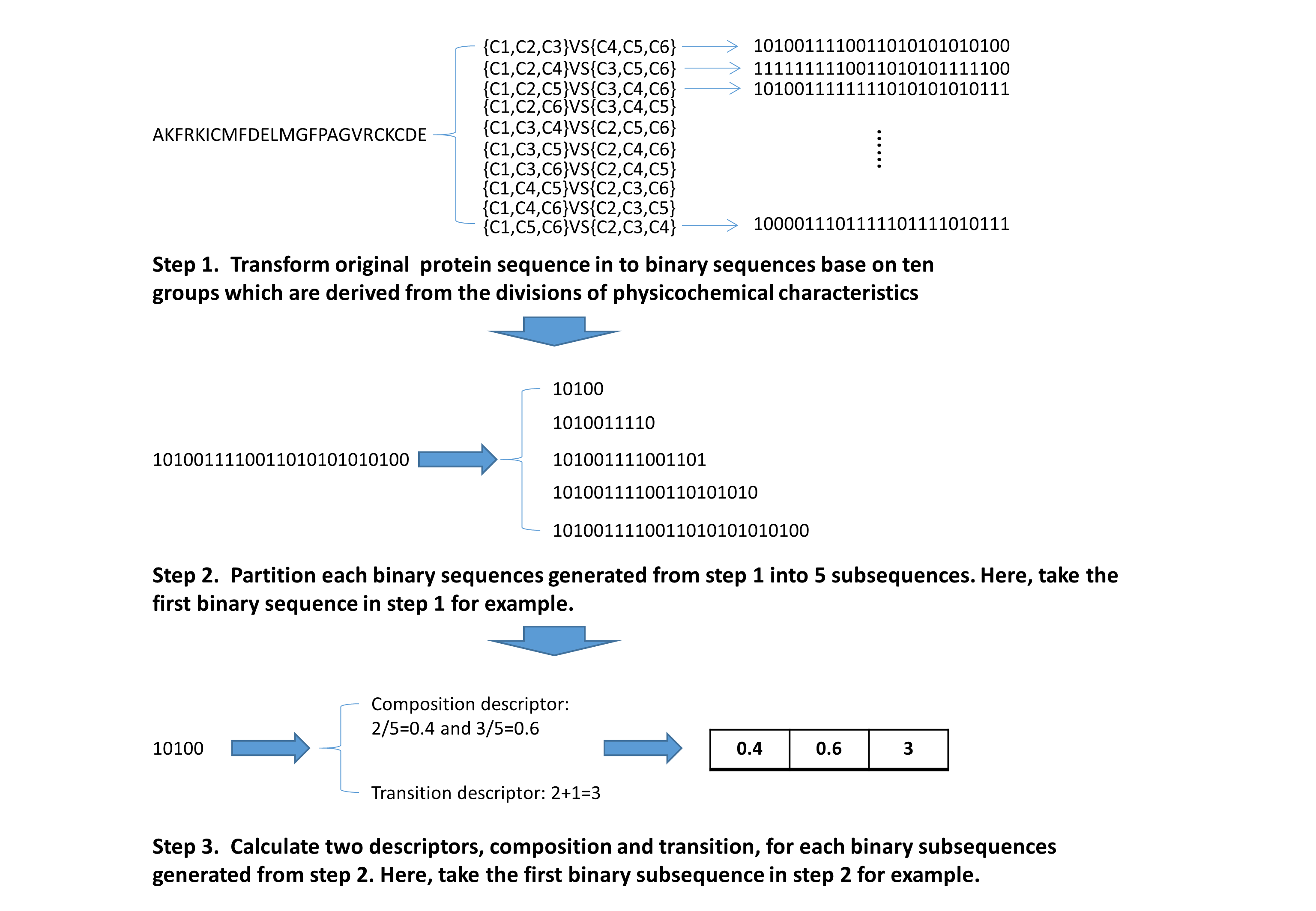

Supplement: Additional file 1: Figure S1. — Example for illustrating the process of global encoding (TIF 781 kb) [file 12859_2016_1035_MOESM1_ESM.tif]

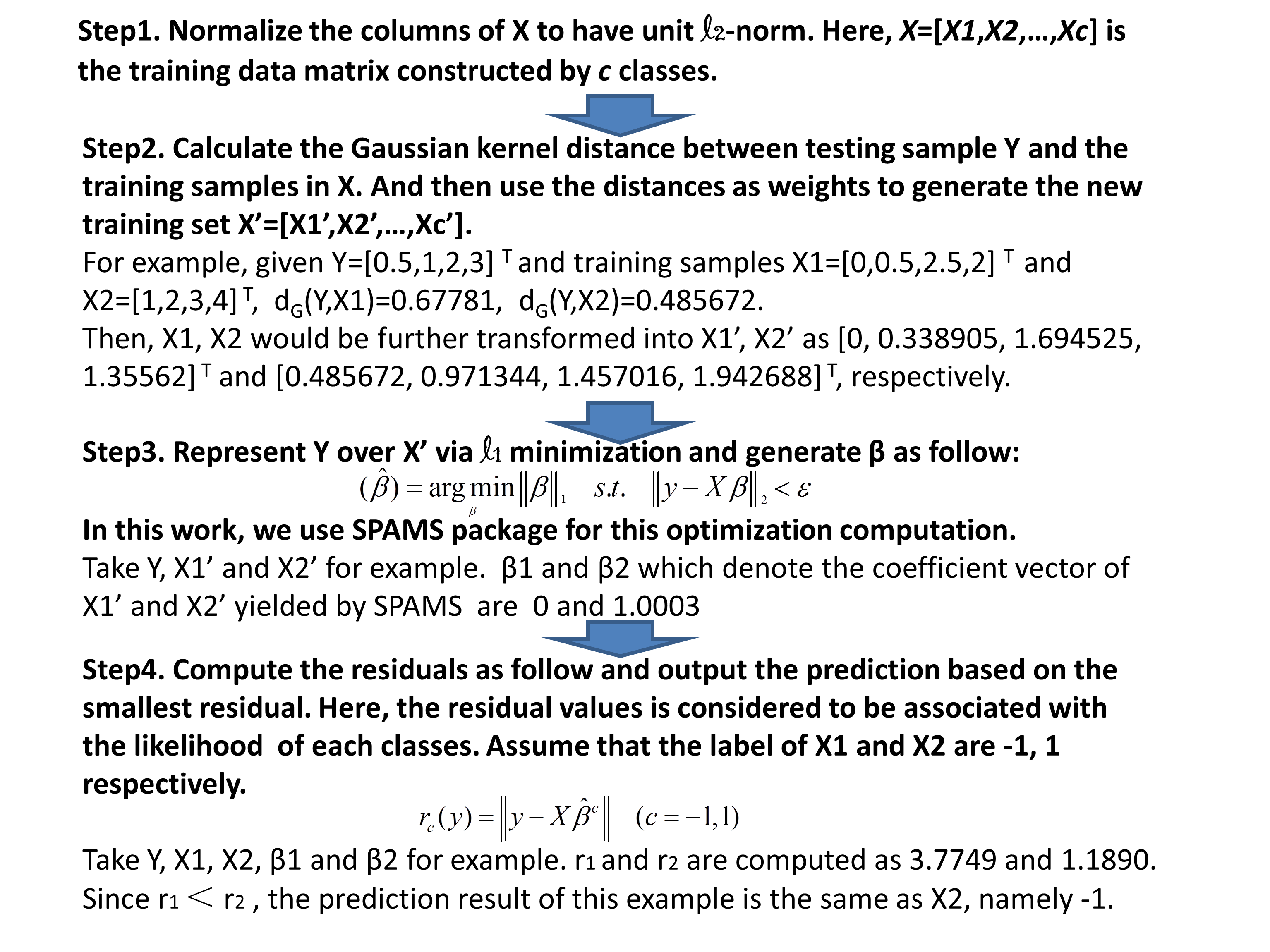

Supplement: Additional file 2: Figure S2. — Example for illustrating the process of weighted sparse representation based classifier. (TIF 1088 kb) [file 12859_2016_1035_MOESM2_ESM.tif]
